# Strain difference in transgene-induced tumorigenesis and suppressive effect of ionizing radiation

**DOI:** 10.1093/jrr/rraa103

**Published:** 2020-11-24

**Authors:** Bibek Dutta, Taichi Asami, Tohru Imatomi, Kento Igarashi, Kento Nagata, Tomomi Watanabe-Asaka, Takako Yasuda, Shoji Oda, Manfred Shartl, Hiroshi Mitani

**Affiliations:** Laboratory of Genome Stability, Department of Integrated Biosciences, Graduate School of Frontier Sciences, The University of Tokyo, 277-8562, Kashiwa, Japan; Laboratory of Genome Stability, Department of Integrated Biosciences, Graduate School of Frontier Sciences, The University of Tokyo, 277-8562, Kashiwa, Japan; Laboratory of Genome Stability, Department of Integrated Biosciences, Graduate School of Frontier Sciences, The University of Tokyo, 277-8562, Kashiwa, Japan; Laboratory of Genome Stability, Department of Integrated Biosciences, Graduate School of Frontier Sciences, The University of Tokyo, 277-8562, Kashiwa, Japan; Laboratory of Genome Stability, Department of Integrated Biosciences, Graduate School of Frontier Sciences, The University of Tokyo, 277-8562, Kashiwa, Japan; Laboratory of Genome Stability, Department of Integrated Biosciences, Graduate School of Frontier Sciences, The University of Tokyo, 277-8562, Kashiwa, Japan; Laboratory of Genome Stability, Department of Integrated Biosciences, Graduate School of Frontier Sciences, The University of Tokyo, 277-8562, Kashiwa, Japan; Laboratory of Genome Stability, Department of Integrated Biosciences, Graduate School of Frontier Sciences, The University of Tokyo, 277-8562, Kashiwa, Japan; University of Wuerzburg, Physiological Chemistry, Biocenter, 97074 Wuerzburg, Germany and the Xiphophorus Genetic Stock Center, Department of Chemistry and Biochemistry, Texas State University, San Marcos, Texas, 78666, USA; Laboratory of Genome Stability, Department of Integrated Biosciences, Graduate School of Frontier Sciences, The University of Tokyo, 277-8562, Kashiwa, Japan

**Keywords:** xmrk, p53, mitf, tumor, medaka, irradiation

## Abstract

Transgenic expression in medaka of the Xiphophorus oncogene *xmrk*, under a pigment cell specific *mitf* promoter, induces hyperpigmentation and pigment cell tumors. In this study, we crossed the Hd-rR and HNI inbred strains because complete genome information is readily available for molecular and genetic analysis. We prepared an Hd-rR (p53^+/−^, p53^−/−^) and Hd-rR HNI hybrid (p53^+/−^) fish-based *xmrk* model system to study the progression of pigment cells from hyperpigmentation to malignant tumors on different genetic backgrounds. In all strains examined, most of the initial hyperpigmentation occurred in the posterior region. On the Hd-rR background, *mitf:xmrk*-induced tumorigenesis was less frequent in p53^+/−^ fish than in p53^−/−^ fish. The incidence of hyperpigmentation was more frequent in Hd-rR/HNI hybrids than in Hd-rR homozygotes; however, the frequency of malignant tumors was low, which suggested the presence of a tumor suppressor in HNI genetic background fish. The effects on tumorigenesis in *xmrk*-transgenic immature medaka of a single 1.3 Gy irradiation was assessed by quantifying tumor progression over 4 consecutive months. The results demonstrate that irradiation has a different level of suppressive effect on the frequency of hyperpigmentation in purebred Hd-rR compared with hybrids.

## INTRODUCTION

Fish models have played an integral role in studying induced and spontaneous melanoma [1]. One of the oldest models of melanoma is the Xiphophorus fish. Hybrids of melanistically pigmented platyfish (*Xiphophorus maculatus*) and nonpigmented swordtails (*Xiphophorus helleri*) spontaneously develop melanoma in melanin-producing cells (macromelanophores) [2]. The melanomas in these hybrids contain a high level of undifferentiated actively proliferating melanocytes [3] and in this respect, resemble the histopathology of human melanomas, which also consist of poorly differentiated melanocytes [4].

Positional cloning of the tumor gene revealed that a mutated epidermal growth factor receptor gene (*egfr*) named *xmrk* (Xiphophorus melanoma receptor kinase), which originated from tandem duplication of the Xiphophorus EGFR, is responsible for the tumorigenesis [5]. *Xmrk*-induced signaling promotes antiapoptosis, proliferation, and cell motility. It stimulates activation of the transcription factor, Stat5, which in turn promotes proliferation and, together with the PI3K pathway, inhibits apoptosis in a manner similar to its effects in human melanoma [6].

Xiphophorus is a live-bearing fish that use internal fertilization and give birth to live young instead of laying eggs for which some biomolecular and genetic techniques have been difficult to apply for further studies on melanoma biology. Although medaka does not have a naturally occurring melanoma system, it offers all the advantages of small-fish experimental models. Apart from its ease of breeding, transparent embryos and suitability for transgenic techniques, an important feature of this fish is the availability of genetically uniform strains [7]. A number of highly inbred strains derived from different natural populations or laboratory fish stocks have been established. This allowed the development of medaka melanoma models [8] in several strains (Carbio, CabR′, albino (i-3) and HB32C) by introduction of the oncogene *xmrk* and use of the pigment-specific promoter *mitf* from medaka to drive its expression [9]. Recently, Sugiyama et al. [10] compared melanocytic tumors occurring in the Carbio and HB11A strains of *xmrk*-transgenic medaka at 7 months post hatching and confirmed a difference in the pathological features of melanoma formation that was dependent on the genetic background.

In this study, we selected Hd-rR and HNI inbred strains of medaka to evaluate genetic background-dependent melanoma formation. The Hd-rR and HNI whole genomes are sequenced and readily available for molecular and genetic analysis [11]. The body color of the Hd-rR strain is white in females (*b/b*, *Xr/Xr*) and orange-red in males (*b/b*, *Xr/YR*), while the HNI strain does not exhibit color differences between sexes. Both these strains are different from the Carbio strain (*B*′*/B*′), which has been widely used for transgenic melanoma model studies and has a variegated melanocyte distribution on its body surface [12].

Whole genome sequence alignments of the inbred strains, Hd-rR and HNI, which were derived from the southern and northern Japanese groups, respectively, indicated that the genome-wide single-nucleotide polymorphism (SNP) rate was 3.42%, and the rate of nonsynonymous SNPs to synonymous SNPs was 0.413 [11]. However, the long-term geographical isolation of these two populations has not resulted in reproductive isolation; they can mate and their F1 hybrids are normal and fertile. We reported the results of expression analysis of Hd-rR and HNI alleles of randomly selected genes using the F1 hybrid and suggested that a considerable number of genes display differential allelic expression in F1 hybrids [13]. In this study, we compared the cancer formation process between purebred Hd-rR and Hd-rR/HNI hybrids heterozygous for a p53 mutation and identified suppressive effects of irradiation on *Xmrk*-induced tumorigenesis.

## MATERIALS AND METHODS

### Fish culture and handling

Fish were handled and maintained using appropriate and ethical research protocols. All fish were maintained indoors under similar optimum conditions. The temperature was maintained at 26–28 °C, under a cycle of 14 h light and 10 h dark phases.

A Cab strain carrying the *mitf:xmrk* transgene was backcrossed more than five times with p53^−/–^Hd-rR fish (Y186X) to produce Hd-rR xmrk^+/−^p53^−/−^ fish. These were crossed with Hd-rR (*xmrk^−/−^p53^+/+^*) or HNI (*xmrk^−/−^p53^+/+^*) fish to obtain the subsequent generations of hybrids with *xmrk^+/−^p53^+/−^* genotypes.

### Genotyping of fish by sequencing and PCR

Genomic DNA was extracted from the caudal fin using the fin clip method [14]. The *xmrk* PCR primers, 5′–TTGGAGCACTACAGCAAGGG–3′ and 5′–GGAAGGAGAGGTCCTGGTTC–3′, were used as forward and reverse primer, respectively to identify *mitf:xmrk* heterozygous fish. For p53, the forward PCR primer, 5′–AAACGAGGACTGTAAGGAAAAC–3′ and reverse primer, 5′–TTGGCTGAAAACAGCACAAC–3′, were used. Sequencing of the PCR products was conducted using the 3500 Genetic Analyzer (Applied Biosystems, Hitachi, Tokyo, Japan).

### Irradiation

Fish were placed in a plastic tube with water and irradiated with gamma rays emitted by ^137^Cs (1.3Gy, Gammacell, MDS Nordion, Ottawa, Canada). The absorbed dose was monitored by InLight® nanoDot™ dosimeters (Nagase-Landauer, Ltd., Tsukuba, Japan).

### Histological sectioning and hematoxylin/eosin staining

The tumor tissues were dissected and fixed in 4% paraformaldehyde at 4 °C for 24 h. After dehydration in ethanol, the fixed tumor samples were embedded in paraffin (Parabett, Lot No. 160101; Muto Pure Chemicals Co., Ltd., Tokyo, Japan). Sections were observed under a light microscope after staining.

### Statistical analysis

Statistical analysis was done by comparing *P* values (≤0.05) that were calculated using the Student’s *t*-test and Fisher’s exact test in Excel® (Microsoft, Redmond, WA,USA).

## RESULTS

### Hyperpigmentation and tumor formation in the genetic backgrounds of Hd-rR and Hd-rR/HNI hybrids

**Fig. 1.**
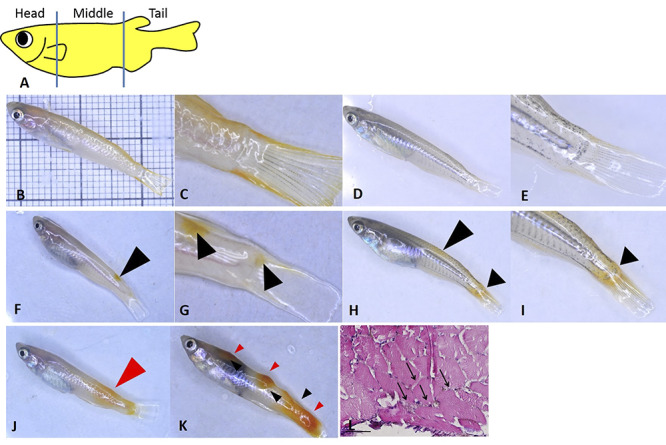
Hyperpigmentation and tumors in Hd-rR and Hd-rR HNI hybrids The body of the fish was divided into three main sections—head, middle, and tail—for the purpose of recording hyperpigmentation and tumors (A).The pigmentation of wild type Hd-rR with no *xmrk* at 2 months (B, C) and of 2-month-old Hd-rR HNI hybrid (D, E). The normal tail region has yellow pigmentation on the sides and the middle of the fin region has a few spots of black pigmentation (C and E). The hyperpigmentation was clearly distinguished from the normal pigmentation by the increase in pigment and the area of pigmentation, which was mainly seen in the tail region. Hd-rR with heterozygous *xmrk* displaying yellow hyperpigmentation in the tail region (F, G; black arrows) and the tail hyperpigmentation of hybrid fish (H, I; black arrows). All tumors develop from hyperpigmentation. The first tumor that develops is termed the primary tumor (J, red arrow), which is small compared to the late phase tumor (K). The metastatic tumors are bigger in size and have nodular growth. *Xmrk* tumors are highly metastatic and invade the muscle tissue beneath the epidermal layer (L, scale, 100 μm). The square mesh (1 mm × 1 mm) used to measure fish size (B).

Hyperpigmentation and tumor formation were observed in *xmrk*-heterozygous Hd-rR and hybrid fish. In both Hd-rR (xmrk^+/−^p53^+/−^; xmrk^+/−^p53^−/−^) and hybrid (Hd-rR/HNI, xmrk^+/−^p53^+/−^) strains, an increase in focal sites of melanocyte proliferation (fish-nevi) development or cutaneous hyperpigmentation (Fig. 1). They can be compared to benign nevi observed in human melanoma Although medaka has four types of pigmentation cells, a prevalence of hyperpigmentation in Hd-rR and its hybrid strain was observed from xanthoerythrophores (yellow hyperpigmentation) and melanophores (black hyperpigmentation) (Tables 1 and 2). The fish body was divided into three broad compartments—head, middle and tail, to assist the quantification of hyperpigmentation and tumors (Fig. 1A). Hyperpigmentation occurred mostly in the tail (data not shown). Indeed, the tail had the highest occurrence of hyperpigmentation compared to the middle and head compartments (Tables 1 and 2).

**Table 1:** Frequency of Hyperpigmentation and tumour in control Hd-rR (non irradiated Xmrk+/−)

p53−/− Hyperpigmentation		Region and color (no. of fish)						Multiple case (no. of fish)	Total no. of fish with case	Total (%)
		Tail			Middle		Head			
Months after hatching	Number of fish observed	Yellow	Black	Yellow	Black	Yellow	Black			
1	15	8	1	3	0	0	0	2	9	60
2	15	11	2	7	0	1	0	6	12	80
3	10	9	4	7	3	4	0	7	10	100
4	8	7	3	6	2	4	0	5	8	100
p53−/− tumor		Region and color (no. of fish)						Multiple case (no. of fish)	Total no. of fish with case	Total (%)
		Tail			Middle		Head			
Months after hatching	Number of fish observed	Yellow	Black	Yellow	Black	Yellow	Black			
1	15	2	0	0	0	0	0	0	2	13
2	15	3	0	2	0	1	0	2	3	20
3	10	6	0	3	0	1	0	2	6	60
4	8	7	0	4	0	1	0	3	8	100
p53+/− Hyperpigmentation		Region and color (no. of fish)						Multiple case (no. of fish)	Total no. of fish with case	Total (%)
		Tail			Middle		Head			
Months after hatching	Number of fish observed	Yellow	Black	Yellow	Black	Yellow	Black			
1	15	4	0	1	0	0	0	1	4	27
2	15	4	1	2	0	1	0	1	6	40
3	15	5	1	3	0	1	0	2	7	47
4	15	5	1	3	0	1	0	2	7	47
p53+/− tumor		Region and color (no. of fish)						Multiple case (no of fish)	Total no. of fish with case	Total (%)
		Tail			Middle		Head			
Months afte hatching	^Number of fish^ observed	Yellow	Black	Yellow	Black	Yellow	Black			
1	15	1	0	0	0	0	0	0	1	7
2	15	3	0	0	0	0	0	0	3	20
3	15	3	0	0	0	0	0	0	3	20
4	15	3	0	0	0	0	0	0	3	20

**Table 2:** Frequency of Hyperpigmentation and tumour in control Hd-rR HNI hybrid (non irradiated, Xmrk^+/−^)

p53+/− Hyperpigmentation		Region and color (no. of fish)						Multiple case (no. of fish)	Total no. of fish with case	Total (%)
		Tail			Middle		Head			
Months after hatching	Number of fish observed	Yellow	Black	Yellow	Black	Yellow	Black			
1	24	16	3	2	0	0	0	1	18	75
2	22	18	10	6	0	0	0	5	20	90
3	22	19	10	8	0	1	0	7	21	95
4	20	18	9	9	0	1	0	8	20	100
p53−/− tumor		Region and color (no. of fish)						Multiple case (no. of fish)	Total no. of fish with case	Total (%)
		Tail			Middle		Head			
Months after hatching	Number of fish observed	Yellow	Black	Yellow	Black	Yellow	Black			
1	24	0	0	0	0	0	0	0	0	0
2	22	0	0	0	0	0	0	0	0	0
3	22	0	0	0	0	0	0	0	0	0
4	20	0	0	0	0	0	0	0	0	0
p53+/− Hyperpigmentation		Region and color (no. of fish)						Multiple case (no. of fish)	Total no. of fish with case	Total (%)
		Tail			Middle		Head			
Months after hatching	Number of fish observed	Yellow	Black	Yellow	Black	Yellow	Black			
1	15	4	0	1	0	0	0	1	4	27
2	15	4	1	2	0	1	0	1	6	40
3	15	5	1	3	0	1	0	2	7	47
4	15	5	1	3	0	1	0	2	7	47
p53+/− tumor		Region and color (no. of fish)						Multiple case (no of fish)	Total no. of fish with case	Total (%)
		Tail			Middle		Head			
Months afte hatching	^Number of fish^ observed	Yellow	Black	Yellow	Black	Yellow	Black			
1	15	1	0	0	0	0	0	0	1	7
2	15	3	0	0	0	0	0	0	3	20
3	15	3	0	0	0	0	0	0	3	20
4	15	3	0	0	0	0	0	0	3	20

**Fig. 2.**
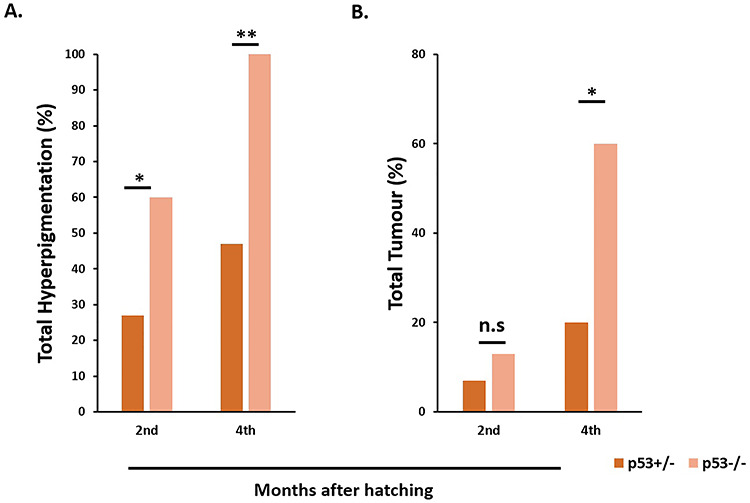
Hyperpigmentation and tumor variation between p53^+/−^ and p53^−/−^ Hd-rR Loss of functional *p53* increases the formation of hyperpigmentation and tumor, as evidenced by the difference in total percentage of hyperpigmentation (A) and tumor (B), which were significantly higher in xmrk^+/−^p53^−/−^ than xmrk^+/−^p53^+/−^ fish (^*^P < 0.05, ^**^P < 0.005, n.s., not significant).

With increasing age, the hyperpigmented regions enlarged from their initial location and new areas of hyperpigmentation began to appear, mostly in the mid dorsal/ventral and head compartments. The earliest onset of hyperpigmentation was seen 1–2 months after hatching; the timing of onset did not change between the genotypes or with p53 status. The frequency of hyperpigmentation differed between p53-heterozygous and -homozygous fish (Fig. 2A and B). In Hd-rR, 60% (9 of 15) of 2-month-old p53^−/−^ fish had hyperpigmentation, while only 27% of the p53^+/−^ fish (4 of 15) developed hyperpigmentation at that age. At 4 months of age, 100% of p53^−/−^ fish (10 of 10) developed hyperpigmentation, but only 47% (7 of 15) of p53^+/−^ fish displayed such lesions (Table 1). In the hybrids, 82% (18 of 22) developed hyperpigmentation at 2 months old, which increased to 100% at 4 months old (Table 2). The other notable difference was in the number of pigment lesions carried by individual fish. Fish that developed more than one hyperpigmented region or tumor were considered to have multiple hyperpigmentation or tumors (Fig. 1J). Notably, significantly more p53-homozygous Hd-rR fish had multiple hyperpigmented lesions than p53-heterozygous fish. At the end of the fourth month, 70% (7 of 10) of p53^−/−^ fish, but only 13% (2 of 15) of p53^+/−^ fish, displayed multiple areas of hyperpigmentation (Table 1, *P* = 0.006). In the hybrids, 32% (7 of 22) displayed multiple areas of hyperpigmentation at 4 months (Table 2). There was no significant difference in the number of hyperpigmented areas between xmrk^+/−^p53^+/−^ Hd-rR fish and hybrids (*P* = 0.14).

Previous studies have revealed that tumor histotypes depend on the genotype of the medaka strain carrying the *mitf:xmrk* transgene [8, 10]. In our experiment Hd-rR displayed formation of either an orange red tumor (xanthoerythrophoroma) or a black tumor (melanoma) or both. Strikingly, no tumor was observed in hybrids (Tables 1 and 2). In Hd-rR all tumors developed from the hyperpigmented regions. The first tumor that developed in the trunk region of adult fish was usually extremely small in size and was termed the primary tumor (Fig. 1I). The tumors were malignant in nature, as they often invaded muscle tissue (Fig. 1K).

### Low-dose gamma irradiation effects on *xmrk*-induced tumorigenesis

**Fig. 3.**
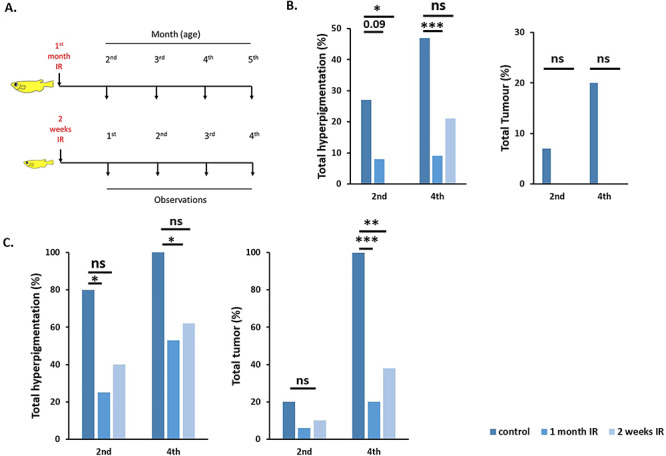
Effects of low-dose gamma radiation (1.3 Gy) on xmrk^+/−^ Hd-rR (p53^−/−^, p53^+/−^) fish at 1 month old and 2 weeks after hatching, with nonirradiated sample results Experimental design of the irradiation experiment. *Xmrk*-mediated hyperpigmentation and tumor formation was observed in medaka after they were irradiated at two different stages of development (2 weeks after hatching and 1 month after hatching). Observations were done during the 4 months following irradiation (A). A reduction in hyperpigmentation and tumor formation was seen after exposure to 1.3 Gy irradiation. A total of 24 p53^+/−^ fish were irradiated 1 month after hatching, and 2/24 (8%) developed hyperpigmentation in the second month, a frequency that did not change in the fourth month (2/23, 9%). A total of 15 p53^+/−^ fish were irradiated 2 weeks after hatching. None developed hyperpigmentation in the second month, but by the fourth month hyperpigmentation increased to 3/14 (21%). No tumor was observed in either 1-month or 2-week irradiated fish (B). For p53^−/−^ fish (C), 16 were irradiated at 1 month after hatching and 4/16 (25%) developed hyperpigmentation by the second month, which increased to 8/15 (53%) in the fourth month. The number of fish with tumor by the fourth month was 3/15 (20%). Fifteen p53^−/−^ fish were irradiated 2 weeks after hatching and 4/10 (40%) developed hyperpigmentation by the second month, which increased to 5/8 (63%). Only 3/8 (38%) fish developed tumor by the fourth month (^*^P < 0.05, ^**^P < 0.005, ^***^P < 0.0005, n.s., not significant).

In studies to investigate the effects of low-dose irradiation on *xmrk*-induced tumorigenesis, fish at 2 weeks and 1 month after hatching were irradiated with 1.3 Gy of gamma rays and the tumor progression was observed until the fourth month and fifth month of age, respectively (Fig. 3A). Larva at 2 weeks after hatching is the late stage of fry, and 1 month of age is before the development of secondary sexual characteristics in fish, so we chose these stages as being corresponding human childhood before puberty.

In Hd-rR, both p53^−/−^ and p53^+/−^ fish displayed significant suppression of hyperpigmented lesions and tumors (Fig. 3B and C). In fish irradiated at 1 month of age, 25% (4 out of 16) of p53^−/−^ fish had hyperpigmentation at 2 months, which increased to 53% (8 out of 15) at 4 months. In p53^+/−^ fish, only 8% (2 out of 24) developed hyperpigmentation during the second month, which only increased to 9% (2 out of 23) at 5 months of age (Table 3). With respect to tumor formation, by the end of the fourth month only 38% (8 out of 15) of p53^−/−^ fish developed a tumor, while no tumor development was observed in p53^+/−^ fish (Table 3). Hd-rR fish irradiated at 2 weeks of age displayed similar results to 1-month-old, irradiated fish (Table 4).

**Table 3:** Frequency of hyperpigmentation and tumour in Hd-rR (Xmrk^+/−^) irradiated (1.3 Gy) at 1 month

p53−/− Hyperpigmentation		Region and color (no. of fish)						Multiple case (no. of fish)	Total no. of fish with case	Total (%)
		Tail			Middle		Head			
Months after hatching	Number of fish observed	Yellow	Black	Yellow	Black	Yellow	Black			
1	16	2	2	2	0	0	0	1	4	25
2	16	3	3	4	0	0	0	2	7	44
3	15	4	3	4	0	1	0	4	8	53
4	13	6	4	4	0	1	0	5	9	69
p53−/− tumor		Region and color (no. of fish)						Multiple case (no. of fish)	Total no. of fish with case	Total (%)
		Tail			Middle		Head			
Months after hatching	Number of fish observed	Yellow	Black	Yellow	Black	Yellow	Black			
1	16	1	0	0	0	0	0	0	1	6
2	16	1	0	1	0	0	0	0	2	13
3	15	1	1	1	0	0	0	0	3	20
4	13	1	1	3	0	0	0	0	5	38
p53+/− Hyperpigmentation		Region and color (no. of fish)						Multiple case (no. of fish)	Total no. of fish with case	Total (%)
		Tail			Middle		Head			
Months after hatching	Number of fish observed	Yellow	Black	Yellow	Black	Yellow	Black			
1	24	1	0	0	1	00	0	0	2	8
2	23	1	0	0	1	0	0	0	2	9
3	23	1	0	0	1	0	0	0	2	9
4	21	3	0	0	1	0	0	1	3	14
p53+/− tumor		Region and color (no. of fish)						Multiple case (no of fish)	Total no. of fish with case	Total (%)
		Tail			Middle		Head			
Months afte hatching	^Number of fish^ observed	Yellow	Black	Yellow	Black	Yellow	Black			
1	24	0	0	0	0	0	0	0	0	0
2	23	0	0	0	0	0	0	0	0	0
3	23	0	0	0	0	0	0	0	0	0
4	21	0	0	0	0	0	0	0	0	0

**Table 4:** Frequency of hyperpigmentation and tumour in Hd-rR (Xmrk^+/−^) irradiated (1,3 Gy) at 2 weeks

p53−/− Hyperpigmentation		Region and color (no. of fish)						Multiple case (no. of fish)	Total no. of fish with case	Total (%)
		Tail			Middle		Head			
Months after hatching	Number of fish observed	Yellow	Black	Yellow	Black	Yellow	Black			
1	15	3	0	3	0	0	0	2	4	26
2	10	3	1	2	0	0	0	2	3	40
3	10	4	1	3	1	1	1	5	5	50
4	8	4	1	3	0	0	2	4	5	62
p53−/− tumor		Region and color (no. of fish)						Multiple case (no. of fish)	Total no. of fish with case	Total (%)
		Tail			Middle		Head			
Months after hatching	Number of fish observed	Yellow	Black	Yellow	Black	Yellow	Black			
1	15	0	0	0	0	0	0	0	0	0
2	10	1	0	0	0	0	0	0	1	10
3	10	2	0	0	0	0	1	0	3	30
4	8	2	0	0	0	0	1	0	3	38
p53+/− Hyperpigmentation		Region and color (no. of fish)						Multiple case (no. of fish)	Total no. of fish with case	Total (%)
		Tail			Middle		Head			
Months after hatching	Number of fish observed	Yellow	Black	Yellow	Black	Yellow	Black			
1	15	0	0	0	0	0	0	0	0	0
2	14	0	0	0	0	0	0	0	0	0
3	14	0	0	0	0	0	0	0	0	0
4	14	1	2	0	0	0	0	1	3	21
p53+/− tumor		Region and color (no. of fish)						Multiple case (no of fish)	Total no. of fish with case	Total (%)
		Tail			Middle		Head			
Months afte hatching	^Number of fish^ observed	Yellow	Black	Yellow	Black	Yellow	Black			
1	15	0	0	0	0	0	0	0	0	0
2	14	0	0	0	0	0	0	0	0	0
3	14	0	0	0	0	0	0	0	0	0
4	14	0	0	0	0	0	0	0	0	0

**Fig. 4.**
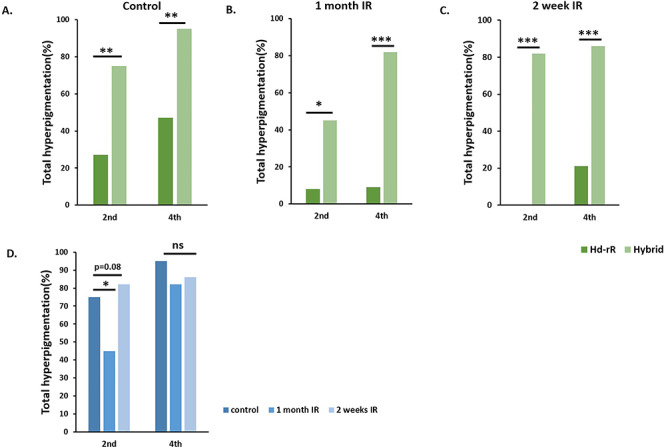
Effects of low-dose gamma radiation (1.3 Gy) on xmrk^+/−^ hybrid (Hd-rR/HNI) (p53^+/−^) fish at 1 month old and 2 weeks after hatching The frequency of hyperpigmentation seen in hybrid fish was higher than Hd-rR. For nonirradiated fish in the second month, 4/15 Hd-rR (27%) had hyperpigmentation and by the fourth month hyperpigmentation had increased to 7/15 (47%). In the case of hybrid fish, 18/24 (75%) had hyperpigmentation in the second month, which increased to 21/22 (95%) in the fourth month (A). The same increase in the frequency of hyperpigmentation was seen in irradiated hybrids. Eighty-two percent (14/17) of 1 month and 19/22 (86%) of 2-week irradiated hybrids had hyperpigmentation by the fourth month, which was significantly higher than in the irradiated Hd-rR. (B, C). In hybrid fish, irradiation at 1 month old was able to induce suppression at both the second and fourth month, but a significant decrease was only seen in the second month. Irradiation at 2 weeks old produced some suppression in the fourth month, but it was not significant (D). Hybrids have different pigmentation and genetic background, and these data indicate that *xmrk* tumorigenesis depends on those factors. The fact that most fish retained the benign phase of tumor progression supports the dependence on *p53* of tumor frequency and the progression from the benign to the malignant phase. (* *P* < 0.05, ** *P* < 0.005, *** *P* < 0.0005, n.s., not significant).

A comparison between purebred Hd-rR and hybrids was conducted for p53^+/−^ fish. The frequency of hyperpigmentation was higher in hybrids (Fig. 4A–C). Hybrids irradiated at 1 month of age displayed a significant reduction in hyperpigmentation, but fish irradiated at 2 weeks post hatching displayed no significant reduction (Fig. 4D). At 2 months, 45% (9 out of 20) of the hybrids developed hyperpigmentation, which increased to 82% (14 out of 17) by the end of the fourth month (Table 5). In fish irradiated at 2 weeks of age, 82% (18 out of 22) developed hyperpigmentation, which increased to 86% (19 out of 22) by the end of the fourth month (Table 6).

**Table 5:** Frequency of hyperpigmentation and tumour in Hd-rR HNI hybrid (Xmrk^+/−^) irradiated (1.3 Gy) at 1 month

p53+/− Hyperpigmentation		Region and color (no. of fish)						Multiple case (no. of fish)	Total no. of fish with case	Total (%)
		Tail			Middle		Head			
Months after hatching	Number of fish observed	Yellow	Black	Yellow	Black	Yellow	Black			
1	20	9	2	0	0	0	0	0	9	45
2	19	13	7	3	0	0	0	3	13	68
3	17	14	8	5	0	0	0	5	14	82
4	17	14	8	6	0	2	0	6	15	88
p53+/− tumor		Region and color (no. of fish)						Multiple case (no. of fish)	Total no. of fish with case	Total (%)
		Tail			Middle		Head			
Months after hatching	Number of fish observed	Yellow	Black	Yellow	Black	Yellow	Black			
1	20	0	0	0	0	0	0	0	0	0
2	19	1	1	0	0	0	0	0	1	5
3	17	1	1	0	0	0	0	0	1	6
4	17	1	1	1	0	0	0	0	2	12

**Table 6:** Frequency of hyperpigmentation and tumour in Hd-rR HNI hybrid (Xmrk^+/−^) irradiated (1.3 Gy) at 2 weeks

p53+/− Hyperpigmentation		Region and color (no. of fish)						Multiple case (no. of fish)	Total no. of fish with case	Total (%)
		Tail			Middle		Head			
Months after hatching	Number of fish observed	Yellow	Black	Yellow	Black	Yellow	Black			
1	22	15	5	0	0	0	0	0	15	68
2	22	18	6	5	0	0	0	5	18	82
3	22	19	9	9	0	4	0	11	19	86
4	22	19	9	11	0	4	1	11	19	86
p53+/− tumor		Region and color (no. of fish)						Multiple case (no. of fish)	Total no. of fish with case	Total (%)
		Tail			Middle		Head			
Months after hatching	Number of fish observed	Yellow	Black	Yellow	Black	Yellow	Black			
1	22	0	0	0	0	0	0	0	0	0
2	22	0	0	0	0	0	0	0	0	0
3	22	0	0	0	0	0	0	0	0	0
4	22	0	0	0	0	0	0	0	0	0

## DISCUSSION

We have demonstrated that in medaka a considerable strain difference exists in transgene-induced hyperpigmentation and tumorigenesis and we identified a suppressive effect of ionizing radiation on the development of the pigment lesions. Previously, it was reported that *xmrk* tumorigenesis in various transgenic lines of medaka depends strongly on the genetic background of the strains. In the Carbio strain, the oncogene was able to induce tumors of the melanocyte and the xanthophore/erythrophore pigment cell lineages, with marked dosage effects on onset of hyperpigmentation and tumorigenesis [8]. In an albino strain, the same oncogenes also gave rise to a high frequency of uveal melanoma. Melanoma rarely developed in the CabR′ strain, in which tumors were mostly xanthoerythrophoroma. Although the majority of tumors in Carbio and CabR′ strains were nodular and exophytic in the HB32C strain, endophytic tumors developed more frequently [15]. In the present study, we observed that tumors that developed in fish of Hd-rR and Hd-rR hybrid background heterozygous for *xmrk*, were exophytic in nature and developed mainly from xanthoerythrophores, while no posterior body skin or uveal tumors were observed. Of note, the frequency of hyperpigmented lesions and tumors depended on the genetic background of the medaka, as reported for *xmrk* in Xiphophorus fish [8].

A comparison of purebred Hd-rR and hybrids revealed that hybrids had a higher frequency of hyperpigmentation than Hd-rR. This result demonstrates that apart from the dosage of the driver oncogene *xmrk*, tumorigenesis in the medaka model is influenced by pigmentation related factors that remain to be elucidated. In both the Hd-rR and hybrid strain, we observed an overabundance of yellow/orange hyperpigmentation.

Kaufman et al. [16] demonstrated that melanoma precursor cells of BRAF^V600E^-transgenic, p53-deficient zebrafish reinitiated an embryonic neural crest signature and activated a melanoma gene program. The niche environment is likely to participate in the process of melanoma initiation and cause the difference in hyperpigmentation and tumor distribution among medaka strains.

The tumor suppressor gene, *p53* is responsible for inducing apoptosis, cellular senescence and cell cycle arrest in response to irradiation and oncogene activation. The role of *p53* is quite evident in the development of melanoma, but *p53* mutations are rare in human melanoma, which may be attributed to the frequent mutations in the *CDKN2A* locus [15]. In melanoma-model mice, oncogenic *Ras* promoted melanoma development in the presence of mutations in the *p53* gene [17, 18]. In a transgenic zebrafish melanoma model, the mitf promoter-driven *BRAF^V600E^* [15] and *N-RAS^Q61K^* [19] genes induced tumors only on a *p53*-deficient background, whereas *H-RAS^G12V^* was capable of tumor formation without *p53* deficiency [20]. In the present study, we found that the age of onset of *xmrk*-induced tumorigenesis (hyperpigmentation and malignant tumor) in the Hd-rR or hybrid strain model did not depend on the *p53* status but rather, quantification of the tumorigenesis process revealed that the frequency of hyperpigmentation and tumors is proportional to the *p53* status. As prior studies have shown that *p53* mutations are not necessary for tumor formation in medaka (different from the situation in the BRAF-transgenic zebrafish), the *p53* status can be better viewed as a tumor modifier rather than a primary driver for melanomagenesis in this fish model.

Radiation may enhance cancer risk [21–23], but studies have shown low-dose radiation-mediated hormesis stimulates repair responses that otherwise remain inactivated, thus imparting beneficial responses to the organism [24]. Long term exposure to low-dose gamma irradiation was able to suppress the tumor incidence in methylcholanthrene-injected mice [25].

An age-dependent susceptibility to radiation-mediated mammary tumorigenesis was observed in a Min mouse model [26]. This study demonstrated that the effect of X-irradiation played a significant role only in the early stages of development in the mice [27]. Thus, we speculate that irradiation exposure at an early stage can influence the probability of tumor occurrence. We demonstrated in this study that hyperpigmentation and tumor development were suppressed by low-dose irradiation in 2-week-old and 1-month-oldfish.

Apoptosis and senescence are integral factors associated with aging [28]. Senescence positive cells increase with age [29] and apoptotic factors are reduced with aging [30]. Whether a balance between apoptosis and senescence based on age exists or not remains to be elucidated. In this study, we examined radiation effects on *xmrk*-induced tumorigenesis and found that a single dose of gamma irradiation administered to juvenile fish can suppress the *xmrk*-induced tumorigenesis process, even after several months irrespective of the status of *p53*.

Previous studies have shown that chronic irradiation has the ability to induce a senescence-like phenotype in vitro [31–33]. A cell enters the state of senescence after crossing the Hayflick limit [34] but ionizing radiation has the ability to induce senescence before the cell reaches the Hayflick limit, a process termed stress-induced premature senescence [35]. The *p53* gene plays an important role in regulating the cell cycle and is an important factor in the induction of radiation-induced senescence and apoptosis [36, 37]. Notably, there are cases where it has been shown that senescence can be induced independently of *p53* [38, 39]. Our results reveal a pause in the tumorigenesis process in both p53^+/−^ and p53^−/−^ fish and we speculate that this involves a role for radiation-induced premature senescence.

This study demonstrates that *xmrk*-transgenic fish with an Hd-rR genetic background can be a powerful tool for understanding the molecular basis of cancer. Polymorphism is an essential characteristic that makes each human unique, with different cancer susceptibility and progression. By crossing genetically different fish strains we can understand how inherited variability influences tumorigenesis and further studies will reveal such natural allelic variation of tumor-modifier genes.

## Funding

This work was supported by the Ministry of Education, Sports, Science and Technology (MEXT) of Japan to H.M. Grant-in-Aid for Scientific Research [1] (25220102) and (18H04135).

## Conflict of interest

There is no conflict of interest between the authors involved in this paper, everyone gave their consent.
